# Franco Basaglia and the radical psychiatry movement in Italy, 1961–78

**DOI:** 10.1332/204986014X14002292074708

**Published:** 2014-08-01

**Authors:** John Foot

**Affiliations:** University of Bristol, UK jf13565@bristol.ac.uk

**Keywords:** anti-psychiatry, deinstitutionalisation, human rights, Italy, Franco Basaglia

## Abstract

This article provides a short introduction to the life and work of Italian radical psychiatrist and mental health reformer Franco Basaglia. A leading figure in the democratic psychiatry movement, Basaglia is little known and often misunderstood in the English-speaking world. This article will seek to address this by highlighting Basaglia’s significant role in the struggle for both deinstitutionalisation and the human rights of those incarcerated in Italy’s asylums during the 1960s and 1970s.

## Biography

Franco Basaglia was born to a well-off family in Venice in 1924. He became an anti-fascist in his teens and was an active member of the resistance during the war in the city. In December 1944, he was arrested and spent six months inside Venice’s grim Santa Maria Maggiore prison. The liberation of the city in April 1945 saw his release. He was briefly a member of the Socialist Party in the post-war period, but never joined another political party.

In 1943, Basaglia signed up to study medicine and surgery in the prestigious and venerable university in nearby Padua. He would later claim that he had chosen his degree subject completely at random. Nonetheless, he was a brilliant student. He graduated in 1949 (despite the war years and his time in prison) and spent the next decade studying philosophy and psychiatry. Despite working in a psychiatric clinic, he was always more interested in ideas and his knowledge of the practical aspects of mental health care was relatively limited. It seemed that Basaglia was destined to have a dazzling university career. But, as is common in Italy (then as now), that institution first used him and then spat him out. He had worked as an assistant to a distinguished professor (Giambattista Belloni) for the entire period from 1949 to 1961, but a real job never materialised. In 1952, Basaglia specialised in the field of ‘nervous and mental diseases’ and in 1958 he qualified as a doctor in that area. But he was eventually told, in no uncertain terms, that he would never be allowed to progress within the university system. He was probably too sharp, too unorthodox, too original, not servile enough, and was advised to look elsewhere for a career. Then, in 1961, a job came up in the town of Gorizia, on the edge of Italy bordering Yugoslavia (today Slovenia). The post was as director of the provincial asylum in Gorizia, which had some 500 patients.^1^ It was an asylum that was similar to many others across Italy (and, in fact, across Europe). Working in an asylum was seen as a dead-end job for psychiatrists at the time – a sign of failure. Gorizia, moreover, was geographically isolated in a series of ways. Nonetheless, Basaglia appeared to have little choice. He took the job.

Basaglia was not a complete outsider, and neither was he a loner. He had friends in high places and he knew how to build alliances and work with those who had power. He also tended to work *within* institutions, and after Padua he would take up positions of authority for himself. Moreover, the philosophical and political ideas he developed in Padua were crucial to his approach to running an asylum after 1961. His life and his career would be marked by radical breaks *and* strong continuities.

Basaglia was director of the asylum in Gorizia from 1961 to 1970, although he effectively moved on from that job in late 1968. From 1970 to 1971 he was director of a large asylum just outside Parma, in a small town called Colorno. In 1971, he took up the same position in Trieste, again on the border with Gorizia. Basaglia remained director of the Trieste asylum until 1979. In that year he moved on to head up provincial mental health services in the province of Lazio, and was based in Rome. However, he was already ill and in 1980 he died of a brain tumour, at the age of just 56. His wife, Franca Ongaro, was at his side (they wrote many of their books together, as joint authors) throughout the Gorizia period in particular. After her husband’s death, she campaigned for the implementation of the so-called ‘Basaglia Law’ – Law 180 – that had been passed in 1978. This law restructured mental health care and called for the closure of all psychiatric hospitals. This process would take at least 20 years to come into effect.

Tall, charismatic and good looking, Basaglia was something of a workaholic. Michele Risso (Risso was a psycho-analyst and a participant in the radical psychiatry movement at the time, as well as a friend of Basaglia) compared him to a ‘big cat’. Once he had power, he fought hard to get his way and could be intolerant towards dissent. He loved to talk, and to argue things out. Occasionally, he could act in an authoritarian manner, and he was stubborn, but he also worked collectively and was aware of the importance of building a team. Basaglia was ambitious and he enjoyed fame and authority, but was completely uninterested in money.

He would usually wake early and work until very late, fuelled by chain-smoking, bottles of coca cola and occasional glasses of whisky. Almost all of his writing (after Padua, in particular) was carried out largely *by and with* his wife.

Many were seduced by Basaglia’s intellect and his personality (including those who had never met him). He was charismatic and charming, and he inspired love and admiration, but also fear, jealousy and sometimes hatred. He became a hero to many, but also an anti-hero for those who were opposed to the movements linked to 1968 (as well as for some who were key figures in ‘1968’ itself). In 1968, he became a symbol for a whole epoch overnight, a household name. A key law was later named after him, a rare honour in Italy, especially for a non-politician.^2^ He was seen as a ‘good man’ but also criticised for what was seen as extreme irresponsibility. He had a strong empathy with his patients, but was blamed by some for abandoning them to their fate. He loved to talk, and to discuss everything, but he could also be intolerant and at times even a little authoritarian. His life was sometimes chaotic, but he never missed an appointment. Work was at the centre of his life. He dedicated himself totally, for nearly 20 years, to ‘the struggle’, and he paid a heavy price for this commitment. Various epithets were applied to him over time, some linked to his well-to-do background and Venetian upbringing: ‘natural leader’, ‘aristocrat’, ‘*patrizio*’ (a nobleman). These were labels used, in the main, by those who did not know him.

## Key concepts and practice

### Gorizia, 1961–69

The post as director of the asylum in Gorizia was distinctly unpromising, and risky. It implied political and geographical isolation, in a sector of the psychiatric system that was going nowhere. Basaglia’s whole family would be uprooted and he would be in charge of a place that had made him feel physically sick. The only point of taking the job was to try to transform the whole system from the edge, from the extreme periphery. He would not simply manage things in the old way, as did most asylum directors at the time in Italy. But there was no clear plan at the beginning, apart from a desire to change things. One advantage of the fact that he was in a dead-end job, in the middle of nowhere, was that nobody expected anything of him. He had a strange kind of freedom he would not have had elsewhere. It would take years even for most Gorizians to notice what was going on their doorstep, let alone people from the rest of Italy.

As director in Gorizia, Basaglia quickly became convinced that the entire asylum system was morally bankrupt. He saw no medical benefits in the way that patients were ‘treated’ inside these institutions. On the contrary, he became convinced that some of the eccentric or disturbing behaviour of the patients was created or exacerbated by the institutions themselves. Although referred to officially as hospitals, these places were very similar to prisons – architecturally and functionally. For the most part, their objective was what Foucault (1991) described as to ‘discipline and punish’.

These convictions were hardened and sharpened by the texts that Basaglia came across in the early 1960s, especially those by Erving Goffman, Frantz Fanon and Michel Foucault. Goffman’s *Asylums: Essays on the social situations of mental patients and other inmates* (1961) unpicked the perverse workings of what he dubbed ‘total institutions’, a phrase that would soon become a key part of the Basaglian lexicon.^3^ Foucault (1961), meanwhile, provided a historical and philosophical focus on the workings of asylums and a theoretical and methodological approach to the study of madness (*Madness and civilization: A history of madness in the classical age*) and the containment of deviance.^4^ Both of these books first appeared in 1961, the year Basaglia took over in Gorizia. The texts circulated in English (and French) before being translated into Italian (in the case of Goffman by Franca Ongaro) in the 1960s.

Inspired by these writings, Basaglia put into practice in Gorizia a series of radical reforms that, by 1968, made the hospital a mecca for activists and one of the capitals of the student movement. These reforms and changes started with the improvement of conditions for patients – an end to restraint, a reduction in electro-shock treatment, the opening up of wards and the destruction of walls and fences. But as time went on, Basaglia introduced more radical change and built up a team (an équipe) of like-minded psychiatrists in Gorizia. General meetings (involving patients from the whole hospital) began in 1965. They became the most public and spectacular part of the Gorizian experiment. Basaglia’s strongest intellectual and personal ally was his wife, Franca Ongaro. She was ever-present in all the struggles in Gorizia.

While Franco Basaglia’s ideas were always linked to the practice of change, they were inspired by a mix of texts and theories. The basis of his work lay in existentialist philosophy – in particular the work of Jean-Paul Sartre. He believed in trying to understand mentally ill patients by building up a relationship with them, and by ‘putting into brackets’ the diagnosis that prevents a proper relationship being formed. As well as Goffman, Basaglia drew on the writings of Primo Levi, and work circulating at the time within phenomenology – Binswanger, Husserl, Minkowski. He was also influenced by the leading theorists of what became known as the ‘anti-psychiatry’ movement – David Cooper, RD Laing, Thomas Szasz. Alongside Foucault and Fanon, Basaglia read work by French psychiatrists such as Lucien Bonnafé. Basaglia and his wife were instrumental in introducing these texts to an Italian audience – in particular Cooper, Goffman and Laing. Franca Ongaro personally translated Goffman’s *Asylums* and Basaglia recommended many of these texts for publication in Italy in the late 1960s and early 1970s.

Following Goffman, Basaglia saw people inside the ‘total institution’ of the asylum as having been reduced to ‘non-persons’ or ‘hollow men’. However, Basaglia brought to this a social analysis of the asylum system. He considered inmates as ‘the excluded’ and a ‘deviant majority’ who had been interned against their will and broken down by the system, although his social critique of the asylum was often crudely drawn (Basaglia divided people into poor people and rich people).

Basaglia also studied the ideas and practices linked to radical psychiatrists working in France, Germany and the United Kingdom (UK). He travelled widely. He was influenced by therapeutic communities he had witnessed or read about first hand, above all through the work of Maxwell Jones in Dingleton in Scotland (a place visited by both Basaglia and his wife, as well as other members of the équipe) and French experiments in institutional psychotherapy. Basaglia not only read the work of, but also discussed his ideas with, ‘anti-psychiatry’ figures such as Cooper, Foucault, Laing and Szasz.

A distinct and specific ‘Basaglian canon’ began to emerge in Gorizia, including philosophical studies and research into the way psychiatric hospitals actually worked. Two collective books were produced by the équipe in Gorizia – *Che cos’è la psichiatria?* (What is psychiatry?) (Basaglia, 1967b) and *L’istituzione negata* (The institution denied) (Basaglia, 1968). The latter was translated across the world (but not into English) and became a bestseller in Italy. Some dubbed it one of the ‘bibles’ of 1968. Both books were hybrid texts, containing theoretical reflections and practical accounts of change in Gorizia, as well as interviews with patients and transcriptions of patient assemblies. These collective books underlined the idea of ‘the institution denied’ whereby radical practice within ‘total institutions’ could overturn power structures within these places, and expose the contradictions within both the system and society as a whole. In addition, these books included powerful descriptions of the conditions within asylums at the time, as well as the class-based structures of the health system as a whole. Finally, however, Basaglia and his team were careful to underline the dangers of these ‘denied institutions’. Therapeutic communities could easily create illusions. They could not resolve society’s problems on their own. Social change was necessary as well as radical reform. Increasingly, towards the end of the 1960s, the language became revolutionary. Maoist slogans also penetrated the movement – although this meant little beyond a series of phrases and a potent idea of the overturning of authority and power.

In summary, three strands to Basaglia’s thought were to take shape over the next 20 years: anti-institutionalism, a social analysis and a biting critique of the medical establishment. But they were all present, in nascent form, right from the start.

After Gorizia, Basaglia spent a brief period in charge of the asylum in Colorno (Parma) and six months in New York where he worked in a psychiatric hospital in Brooklyn. In 1971, he was offered the post of director of the asylum in Trieste.

### The end of the asylum: Trieste 1971–79

Trieste no longer has a psychiatric hospital. In January 1977, Basaglia held a press conference in the city. The news was a simple announcement. San Giovanni psychiatric hospital, the city’s vast asylum, was to be closed by the end of the year. It didn’t quite happen that quickly but the hospital stopped accepting patients in 1980, and soon there were so few patients inside the complex that even to call that institution a ‘hospital’ was clearly incorrect. After just six years as director, Basaglia had achieved the impossible. The institution was not merely ‘negated’, it had been obliterated. The Basaglian movement reached its peak and achieved its moment of greatest fame in Trieste during the 1970s and afterwards. San Giovanni psychiatric hospital can claim to be the first asylum in the world to be closed for political reasons – because those who ran it believed it to be an abdominal place, a concentration camp. The events in Trieste also led directly to a national law – Law 180 – the ‘Basaglia Law’ that called for the closure of *all* Italian asylums. So how did this revolution happen and what is its legacy?

### Phases of closure

Trieste was not Gorizia, and 1971 was not 1961. A vast movement was in full flow across the world and ‘anti-psychiatry’ was a key part of its driving ideology. Once in charge in Trieste, Basaglia and his team moved with great speed. The plan was simple: *to* *close down the hospital*, from above, and quickly. Everything seemed possible. The Gorizian utopia was to become a concrete reality in Trieste. It was as though things were stuck on fast forward.

The contradictions inherent in the Basaglia project – a group of people in charge of an institution that did not believe had a right to exist, and that many of them saw as akin to a Nazi concentration camp – were to be resolved. This time, the doctors would not leave the institution intact. The resolution of the contradiction would take place in another way in Trieste, with the end of the institution itself. The institution was not to be negated. It would be eliminated, forever.

Basaglia moved fast. Between 1971 and 1974, the asylum went through many of the changes that had taken almost double that time in Gorizia. Patients were given back their basic human rights and wards were opened. The rigid spatial gender divisions in the asylum were ended (leading to a series of ‘moral panics’ over ‘intimacy’ between patients). Some steps taken were new ones. The hospital was divided into sectors (corresponding to different areas of the city and province) in preparation for its closure, an idea borrowed from French reformers.

Cooperatives were also set up. This was another new tactic, and allowed for patients to move straight into the world of work. Cooperatives would be widely used across Italy to ‘re-integrate’ mental health patients back into society in the 1970s, 1980s and 1990s. Such ‘sectoral’ reforms allowed the asylum itself to survive, and Basaglia called for the asylum’s ‘destruction’. However, this use of sectoral ‘tactics’ showed how Basaglia was interested above all in practical change and was not dogmatic. He was willing to borrow ideas from a variety of sources as long as they worked.

This period also saw the creation of a vast and multi-layered team (équipe) that had meetings as well as more spectacular parts of the creative strategy, such as street theatre. These were not general meetings across the whole hospital, but smaller encounters that were concerned with policy and strategy. There were also regular ward meetings to which everyone was invited, sometimes on a daily basis.

The period was accompanied by political events linked to the praxis of 1968, such as occupations (of empty council structures) and the push out to permanent territorial centres in various areas and inside the city’s hospital. During the 1970s, the psychiatric hospital grounds in Trieste were transformed into an experimental space, hosting art and theatrical projects, exhibitions, plays, conferences, concerts, numerous debates and meetings and international congresses. Militants, students, intellectuals and practitioners flocked to Trieste. It was a time of extraordinary ferment.

While in some places institutions were replaced with other alternative forms of institution (a process given different labels such as deinstitutionalism and anti-institutionalism), Trieste was one of the places where 1968 was put into practice. The slogans that popped up all over the hospital were those of the movement: ‘*la libertà e terapeutica*’ (‘freedom is therapeutic’) or ‘*la verità* è rivoluzionaria’ (‘the truth is revolutionary’).

Community housing was also set up, at first *inside* the hospital complex itself, as wards were unlocked and closed down. The Trieste experience mobilised thousands of people. Links were forged with the city and strengthened with student activists all over Italy, and internationally. Volunteers began to arrive hoping to work at the site, some from local schools and universities, others from abroad, as well as psychiatrists and medical experts influenced by Basaglian thinking. As one visitor said: ‘Everybody went to Trieste.’ (Crossley, 2006: 3877). For Crossley (2006: 3922): ‘Trieste exerted a “magnetic pull” for radical psychiatrists and animated them.’

As in Gorizia and elsewhere, the so-called deinstitutionalisation of Trieste’s asylum in the 1970s was a constant struggle:

- against the local judiciary;
- against public opinion;
- against the local press (which was extremely hostile to Basaglia and his team);
- in the face of organised political opposition (the neo-fascist party was very strong in Trieste).

However, political protection was provided by the majority in the province. The leading figure, Michele Zanetti, took most of the considerable flak and criticism linked to these reforms. This left Basaglia and his collaborators relatively free from the constant interference that they had experienced (in different ways) in Gorizia and Parma.

There were also constant internal political debates, which would become more and more intense as the 1970s wore on. Trieste had a widely publicised ‘incident’ in 1972 (in fact, there was a whole series of incidents, but only one attained national news status). A patient on day release had murdered his wife in Gorizia in September 1968 (an event often referred to as an ‘incident’), and this had led to a crisis in terms of the Basaglian project. In 1972, an ex-patient murdered his parents in Trieste. This second ‘incident’ had less of an effect as the team in Trieste were far more prepared to deal with the judicial and political fallout that ensued. Nonetheless, these incidents showed the risks that Basaglia and his team were taking. They took full responsibility for what had happened but argued (on both occasions) that the real problem lay with the system itself.

Basaglia and Zanetti filled the hospital in Trieste with doctors, volunteers, psychologists, sociologists, militants, artists and musicians, and emptied it of patients. An incredible 122 people were taken on to work in the asylum under the Basaglia regime. In Gorizia, there had only been six doctors. Ex-patients were now provided with cash benefits and housing. Other patients were ‘volunteers’ who had never been under the previous ‘forced recovery’ regime. Some of these were private patients. Paradoxically, as the number of patients diminished, the number of Basaglian ‘*operatori*’ (workers) increased massively. By the end, there were more *operatori* than patients.

Basaglia was the undoubted leader of this whole experience. He was also more of a one-man band than he had been in the past. Although Franca Ongaro was often around in Trieste, she was based in Venice throughout this time. Her role in Gorizia was much more central than during the 1970s. The couple still worked together on a series of books and projects, but Basaglia also collaborated with others. In Gorizia, the Basaglia family had been an integral part of the experience. In Trieste, the undoubted protagonist and leader was Franco Basaglia.

### Trieste: history and memory

As the 1970s wore on, and right into the 1980s and 1990s, Trieste became a beacon for change. It was the symbol of what could be done, of radicalism in general, of a social, cultural and medical revolution. Much more than Gorizia, Trieste became a ‘concrete utopia’, a place where transformation could be touched, experienced, seen with your own eyes. Basaglia presided over all this with the experience of Gorizia and Parma behind him. He was not interested in creating another ‘golden cage’, or a Maxwell Jones-like therapeutic community. All of that was superfluous, a waste of time. The key work would be outside of the asylum, in the city of Trieste and across the province. It was time not just to break down the walls, but also to construct something entirely new, an alternative to the psychiatric hospital itself. Time would not be lost in internal conflicts with hostile doctors, nurses or administrators. Things were moving firmly in the direction the Basaglians wanted. They had, literally, taken over the asylum.

The general assemblies used in Gorizia were abandoned and replaced with daily open staff meetings that were used to decide on strategy. Much more than in Gorizia, the strategy employed in Trieste reached out way beyond the walls of the asylum. The whole array of ‘the movement’ was employed in order to galvanise public opinion and as part of a sophisticated media strategy, in alliance with artists, theatre directors, actors, musicians, film makers and others. Trieste became a beacon for the Left across Europe and beyond. For example, a number of activists from the anti-psychiatric SPK (Socialist Patients Collective) movement in Heidelberg in Germany, which had been closed down by the authorities, turned up to work in Trieste. Some are still there today. (I do not intend to go into detail about the Heidelberg movement here, or the role of its activists in Trieste. For an account of the SPK, see SPK, 1993.)

When Basaglia had become director in Trieste in 1971, there were 1,182 patients in the psychiatric hospital, 90% of whom were non-voluntary and still held under the provisions laid out in the 1904 law, (this law governed the psychiatric hospital system in Italy until the reforms of the 1960s and 1970s) while the remaining voluntary patients came under the 1968 law. By the beginning of 1977, only 51 patients inside the hospital were being held as ‘forced’ inmates, although there were still many with ‘guest’ (433) or ‘volunteer’ (81) status. In August 1980, nine years after Basaglia’s arrival, Trieste’s asylum closed for good (for another account from the inside, see Dell’Acqua, 1995: 151–5).

Today, Trieste has no psychiatric hospital and Italy itself is without asylums. San Giovanni is used as a park, and houses a school, part of the university, various health services, cooperatives and bars. It is a peaceful and beautiful place, which is an integral part of the city. This alone is a lasting legacy of the movement that began in near-total isolation in 1961 in Gorizia.

Trieste’s hospital was not just closed down, with speed; its whole raison d’être was undermined, built as it was on separation, exclusion and silence. The period of closure was noisy and joyful, and impossible to ignore. From a total institution, built on its own rigid set of rules, violence and the idea of a closed world, the Trieste asylum was first transformed into an open, creative place, a place where freedom and debate were more common than in the outside world, a model for change. It had become an anti-asylum. It is now something else, an *ex*-asylum.

## Critical debates: a missing translation in the English-speaking world?

The history, biography and practice of Franco Basaglia and the *psichiatria democratica* (democratic psychiatry) movement he partly led and inspired has, with a few exceptions, been consistently misinterpreted in the English-speaking world (and in particular in the UK, although one exception is Ramon, 1988). Let us take, for example, the judgements of two of the leading historians of ‘madness’ and ‘asylums’. In 2002, Roy Porter wrote: ‘In Italy, leadership of the movement was assumed by the psychiatrist Franco Basaglia, who helped engineer the rapid closure of institutions (chaos resulted)’ (Porter, 2002: 210). In 1994, Porter referred to Basaglia as ‘Enrico Basaglia’ and labeled him as a ‘boisterous anti-psychiatrist’ (Porter and Micale, 1994: 20). Andrew Scull’s judgement on Basaglia was similarly brief, in 2011: ‘In Italy, led by the charismatic Franco Bassaglia [sic], the political left led the charge’ (Scull, 2011: 113). A more balanced and well-informed account (although with some errors) can be found in Burns (2013: xlvi, 148–9, 183). However, even here, Basaglia is described as a ‘Gramscian Marxist’.

The origins of these snap and inaccurate judgements lie in a series of areas. First, Basaglia’s work was not translated into English, including (and most importantly) *L’istituzione negata* (Basaglia, 1968). This book was, however, quickly translated with success into numerous other languages. There are no convincing explanations of this ‘non-translation’, although there are various accounts available (some claim that Laing himself blocked a translation, but I have found no evidence to back up this claim; www.janushead.org/4-1/jenner.cfm). The non-translation of *L’istituzione negata* became something of an issue for the ex-members of the équipe and perhaps, in particular, for Basaglia. They wanted to have an influence in the English-speaking world, a world that had been an inspiration for them and their practice.

Basaglia’s other writings and those of his team were only translated into English in piecemeal fashion and usually in hard-to-find or largely academic publications, and often well after the events described in his work had taken place. There is a collection/study of his writings edited by Scheper-Hughes and Lovell from 1987, and a short and much-quoted article (Basaglia, 1981) appeared in Ingleby’s (1981) *Critical psychiatry* in the early 1980s. However, the lack of a translation of *L’istituzione negata* was especially important. First, it was the central text of the movement, and it had been influential in France and Germany. English-speaking readers were never given the chance to read it. Second, Basaglia was the subject of a series of extremely hostile but influential studies in English in the 1980s in the wake of the ‘Basaglia Law’ and debates in the UK about closure of asylums, as well as the backlash against ‘anti-psychiatry’ (Jones and Poletti, 1984, 1985). These articles then led directly to critical comments on Basaglia and the ‘Basaglia Law’ in important books about psychiatric reform and the meaning of mental illness, particularly in the light of attempts to regain the ground lost to Laing and the anti-psychiatry movement.

A striking example of this kind of analysis can be found in Roth and Kroll’s (1986) *The reality of mental illness*. This book was intended as a rejoinder to anti-psychiatrists and was widely read at the time. It would appear to be the source for some of the snap and dismissive judgements made by Porter and Scull. Roth and Kroll appeared to be unaware that Basaglia had died in 1980 when they wrote that ‘Basaglia is a Marxist’ (1986: 23/4). They went on to argue that Basaglia’s analysis of mental illness was ‘ideologically driven and very naïve and, in a sense, very callous’. Basaglia was accused in no uncertain terms of throwing asylum inmates onto the streets for political reasons, and Law 180 was described as a ‘disaster’, in social and human terms. The conclusion was that this was what happened when mental patients were ‘exploited … as pawns in an ideological struggle’ (1986: 23–4). Roth and Kroll concluded their comments by giving their support to moves to repeal Law 180.

Roth and Kroll’s trenchant criticism took its cue, in turn, from a notorious article published by Jones and Poletti (1985) in the *British Journal of Psychiatry*. This article was six pages long, and led to a major debate in the journal, including a flurry of critical letters. In their article, Jones and Poletti set out to analyse what they called the ‘Italian experience’. They defined this as the implementation of Law 180, which was passed in 1978, and made only perfunctory reference to what had happened before that date. The only Basaglian text examined in any detail was a talk Basaglia had given in the UK in 1979. The authors claimed that the passing of Law 180 had been seen as one of the ‘great success stories of psychiatric history’ in the UK, and that they wanted to present a more balanced picture. Their study was based on research into published sources and a ‘study tour’ of Italy in 1984. On this ‘tour’ they had visited a series of mental health institutions, ‘chosen at random’. They claimed that Law 180 had lost support, and was due to be repealed (as I write, in 2014, this has not happened). The final part of the article examined what the authors called ‘the negative effects’ of the law. (This section involved a series of somewhat random quotes from the press, many of which had their titles misspelt.)

It is beyond the scope of this article to examine in detail the effects of the ‘Basaglia Law’. However, what is interesting for us, here, is the way in which this law was blamed for a whole series of problems on the basis of flimsy evidence, and that part of this blame was transferred back to the ideas and practice of Basaglia himself. But this was at least nuanced to some degree. As Jones and Poletti (1985: 347) wrote:

A third reason [for the failures of Law 180] is a possible confusion between the thought of Franco Basaglia, the current aims of Psichiatria Democratica, the intention of Law 180, and the outcome. The politico-social theory, the pressure-group campaign, the legislative provision and the state of the services seven years later are causally and temporally linked, but not identical. Basaglia, who cared about the condition of his patients, might have taken a very different view in 1985 if he had lived.

Jones and Poletti’s 1985 article led to something of an outcry (see, for example, Saraceno, 2012), and they were forced into a clarifying article in 1986. This involved further trips to Italy, and this time they visited Trieste. In this second article, the picture they painted was detailed and positive (about Trieste). But they also argued that the hospital had not really been ‘closed’ at all and questioned the real content of services in the city (Jones and Poletti 1986; in contrast, see also Lovestone, 1985, 1988; Ramon, 1985a, 1985b; Tansella, 1986).

There was wide-ranging debate among practitioners, activists and researchers in the UK about the Basaglian experience and especially about the impact of Law 180, with both positive and negative evaluations of the Italian case, but only one side of this debate appears to have been picked up by many commentators. It is not true that reaction in the UK to the law and its aftermath was universally negative, but it does seem to be the case that it is the negative aspects and arguments that have survived the debate, while the other points and discussions have been forgotten or marginalised. Thus, it becomes possible that Basaglia can be simply dismissed as an anti-psychiatrist and his reforms equally dismissed as simply leading to ‘chaos’. While it is clear that many activists and practitioners were inspired by the Basaglian experience, and especially by Trieste, the historical discussions that have followed have not, with very few exceptions, taken this into account (athough for an exception, see Crossley, 2006). The lack of key texts in English, especially *L’istituzione negata* (The institution denied; Basaglia, 1968) and *Che cos’è la psichiatria?* (What is psychiatry?; Basaglia, 1967b) certainly impoverished the debate that took place.

These comments and the focus of the discussion probably led to the lapidary and dismissive conclusions by Porter and Scull. This article is, in part, an attempt to correct this interpretation, and provide the Basaglian movement with historical background and content from the period *before* the 1978 law was passed. (There is also a consistent strand of anti-Basaglia literature in the academic world. For example, Romanucci-Ross and Tancredi, 2007: 11, describe Law 180 and the Basaglia movement as an ‘experiment which failed’ and a ‘great cultural error’.)

## Conclusion: the story of a movement

1978 saw the passing of Laws 180 and 883, which set up Italy’s national health service and eventually led to the closing down of all psychiatric hospitals. In 1980, Basaglia died of a brain tumor. He was just 56 and would not see the reforms he had inspired put into practice. Franca Ongaro (Basaglia’s wife), carried forward the struggle to implement these laws. It was a long and difficult battle, and there were numerous attempts to block or simply ignore the reforms. In the end, however, that battle was won.

The story of the radical movement within (and outside) psychiatry began in Gorizia in the early 1960s and then moved on to a whole series of other places – Arezzo, Parma, Perugia, Reggio Emilia, Trieste.

A small group of young and radical psychiatrists, led by Basaglia in Gorizia and by others in different cities, simply refused to accept the state of affairs they had come across inside asylums across Italy. In their push to change things, these psychiatrists were aided and abetted by nurses, volunteers and above all (in some places) by a new class of administrators and politicians. This post-war political class wedded itself to the desire for a new kind of psychiatry and for the transformation (and eventually closure) of the old asylum system. They were not driven by greed, or the desire for power. Humanistic principles and a moral imperative (these places were simply not acceptable) pushed them to press for reform.

This was a collective ‘no’. And this ‘no’ changed the world. It was not acceptable to treat people in that way – without rights, without autonomy, without knives and forks, without hair, without any control over their own treatment. It was wrong to electrocute these people, cut out bits of their brains or tie them up for years on end. This movement was a struggle for liberation, for democracy and for equality. These 100,000 inmates of mental asylums had disappeared from history.

They needed to re-emerge – to be given back their own identity and dignity. This generation of politicians and psychiatrists was a post-war, anti-fascist generation. There was something profoundly anti-fascist about the anti-asylum movement. It was a movement about human rights. The people inside the asylums were *people*.

The other protagonists of this story, therefore, are the patients themselves. They were also part of the movement, although they have rarely been seen as such: patients such as Carla Nardini in Gorizia – who had been in Auschwitz – or Mario Furlan (also in Gorizia, and who later committed suicide. These people had their lives changed by the revolution in psychiatric care, but they also retook control *of* their own lives. Without them, the movement would never have even begun to have an effect.

Italy did not see the emergence of a real patients movement on the back of the Basaglia experience. The numerous cooperatives that were used to absorb and reintegrate thousands of patients back into the world of work were the closest to the UK experience of a service users movement. However, Trieste had an enormous influence on ‘*operatori*’ and patients movements in the UK and elsewhere. It became a kind of ‘practical utopia’ for many and was at the centre of debates in the UK and elsewhere. It showed what was possible or, as Basaglia put it, that ‘the impossible was possible’ (Basaglia, 2000: 142).

The movement had its beginnings in Gorizia, but its scope and reach went far beyond the story of Franco Basaglia and Franca Ongaro. As one psychiatrist from the time has argued: ‘the transformation of Italian psychiatry was as a result of a polycentric movement’ (Giacanelli, 2008). The Basaglias were crucial – central – to the movement for change.

Every city, every asylum, carried forward its own version of change. Along the way, great risks were taken. Some people were murdered, others committed suicide. Families had to deal with sons, daughters, mothers and fathers who had serious problems, and who had been shut away behind closed doors for years. The outside world was a difficult place in so many ways. It was easy for ex-patients to fall through the cracks in society. Once the enemy of the asylum had been abolished, the real work began. As Forgacs (2014: 404) has written: ‘The story of psychiatric reform in Italy did not end with the passing of Law 180 in May 1978. On the contrary, the most difficult phase of the movement for reform began when the law came into force.’

Undoubtedly, the movement was also marked by numerous ‘excesses’ of ideology, exaggerations, the use of inflammatory and dangerous language, simplifications and dogmatisms, sectarianism and bitter disputes over what seems, today, to be very little indeed. These excesses were often taken up by the followers of the movement, whose sloganeering and empty phrase making did little to help those with mental health problems in the real world. Basaglia himself was aware that mistakes had been made. Often, the language used by the movement provided the movement’s enemies with ready ammunition. Maoist slogans were common. The envelope was frequently pushed too far. Too often, a problematic link between social class and mental illness was drawn or simply stated – as if it was an obvious fact. In the heady and violent times of the 1970s, ‘traitors’ were easily identified and dismissed. The movement was riven by conflict, personal division and hyperbole. Only in retrospect can we pick through the embers of what happened and try to bring some order. It was a time of excess. The revolution appeared to be around the corner. It wasn’t.

The final key component of the movement were the fellow-travellers – intellectuals, writers, film makers, journalists, photographers and artists – who gave up their time and their talents in order to press for change. These people were central to the success of the movement, as they provided a connection between the high theorising of the leaders and the masses. This was true of publishers, television and film producers and directors, artists and theatre directors, photographers. When more than 10 million people saw patients from Gorizia’s psychiatric hospital speaking to presenter Sergio Zavoli on their television screens, inside their own homes, in January 1969, the movement was given a push, which it would never have had by any other means.

Today, Italy’s former asylums perform a variety of functions. Some are empty and abandoned. Others are ‘museums of the mind’. Many still have links to health and mental health services. Some are schools, some are universities, some have become housing. Most are now beautiful parks, at least in part. The ‘great confinement’ described by Foucault (2009: 44) gave way, in the 1970s, to a ‘great liberation’. Society absorbed most of the 100,000 inmates who had been kept inside the places. This process was forced on the system by a movement that acted from inside the institutions themselves, in a way that was unique in the Western world. Italy’s asylums were closed down by the people who worked inside them. In doing so, these people abolished their own jobs – forever. Nobody, today, is the director of a psychiatric hospital in Italy. The movement acted against its own self-interest – in a way that was the opposite of clientelism, patronage and nepotism. It was a negation of itself.

Much of what was called for in the heady days of the movement never came to pass. The interest in radical psychiatry began to fade, and the backlash began in earnest. The movement ended up on the defensive – clinging onto the gains of the 1960s and 1970s. As one protaganist wrote in 1969:

We were looking for an alternative to psychiatry: we were experimenting and trying out new ways of doing things. In our society, an alternative form of psychiatry was only possible in part, and only for a brief time. Afterwards, above all in places where it worked, it became ‘dangerous’ and then it was repressed or integrated, neutralised. All of this was inevitable and we knew that it was so, but we all learnt a lot during this long march. (Jervis, 1969: 259)

It is not easy to write about this movement, with its myths, splits, silences and possessive memories. There was also something about the movement itself that created problems in terms of memory. A version of that past does exist – but it is largely celebratory. The prevailing, public versions of the Trieste–Basaglia story tend, however, to simplify the past.

Contemporary debates around Basaglia’s reforms and ideas tend to concentrate in two areas. The first is linked to the closure of the asylums, and the alternative structures that were set up in various countries (as well as Italy) to ‘replace’ them. A considerable body of opinion claims that the ‘Basaglia Law’ was a mistake, which ‘abandoned’ patients and failed to create adequate alternative structures. But there are many parts of Italy with excellent services that are still the envy of the world. A second set of debates is linked to the movement itself, and its excesses. Here there is a tendency to mythologise, on the one hand, and demonise, on the other. In the end, this was a complex and contradictory movement, which opened up a series of questions and mobilised large numbers of psychiatrists, activists and patients, as well as nurses and volunteers. The ‘Basaglia Law’, for all its limitations and inadequacies, remains one of the great examples of reform linked to radical practice and theory from the 1960s and 1970s.

## Figures and Tables

**Table 1:** Numbers of patients (without ‘guest’ status) in the San Giovanni psychiatric hospital, Trieste, 1971–78

Year	Number of patients
1971	1,182
1972	1,058
1973	930
1974	625
1975	470
1976	253
1977	132
1978	87

## Notes

1 The terms ‘patient’, ‘mental illness’ and ‘mentally ill’ are used in this article. This reflects the dominant terminology in use at the time of Basaglia’s interventions.

2 Yet this identification of the law with one man was an historical error, which downplayed the strength and variety of the movement that led to the reforms. In recent times it has become common practice in Italy to name unpopular laws after those promoting them in Parliament.

3 Goffman’s work is present in the first collective book to come out of Gorizia – Basaglia (1967a) – and permeates the second collective book – Basaglia (1968). Franca Ongaro translated Goffman’s *Asylums* for Einaudi and the book came out in the same series as Basaglia (1968) just three months after the Gorizia book, with a preface written by Franco and Franca Basaglia (Goffman, 1968). For Goffman and Basaglia, see d’Alessandro (2008).

4 Parts of this book were read in Italian by the Gorizian équipe. The Milanese publisher Rizzoli first translated and published the book in Italian in 1963: Foucault (1963). Basaglia had also read Foucault’s introduction to the French translation of Ludwig Binswanger’s *Traum und esistenz* (Foucault 1954] in the 1950s, according to Pirella (1998). For Basaglia and Foucault, see Di Vittorio (1999) and (*aut aut*, 2011). Babini (2009: 245) argues that A history of madness became ‘alla fine degli anni Sessanta un testo sacro’ (‘by the end of the sixties … a sacred text). In 1969, Einaudi published *Nascita della clinica* (*Birth of the clinic*) (Foucault, 1969).
